# Carnitine and/or Acetylcarnitine Deficiency as a Cause of Higher Levels of Ammonia

**DOI:** 10.1155/2016/2920108

**Published:** 2016-02-21

**Authors:** Cecilia Maldonado, Natalia Guevara, Cecilia Queijo, Raquel González, Pietro Fagiolino, Marta Vázquez

**Affiliations:** ^1^Pharmaceutical Sciences Department, Faculty of Chemistry, Universidad de la República, Avenida General Flores 2124, P.O. Box 1157, 11800 Montevideo, Uruguay; ^2^Newborn Screening Laboratory, Social Security Institute, Tristán Narvaja 1716, 11200 Montevideo, Uruguay; ^3^Toxicology Department, Hospital de Clínicas, Universidad de la República, Avenida Italia s/n, 11609 Montevideo, Uruguay

## Abstract

Blood carnitine and/or acetylcarnitine deficiencies are postulated in the literature as possible causes of higher ammonia levels. The aim of this study was to investigate if the use of valproic acid, the age of the patients, or certain central nervous system pathologies can cause carnitine and/or acetylcarnitine deficiency leading to increased ammonia levels. Three groups of patients were studied: (A) epileptic under phenytoin monotherapy (*n* = 31); (B) with bipolar disorder under valproic acid treatment (*n* = 28); (C) elderly (*n* = 41). Plasma valproic acid and blood carnitine and acyl carnitine profiles were determined using a validated HPLC and LC-MS/MS method, respectively. Blood ammonia concentration was determined using an enzymatic automated assay. Higher ammonia levels were encountered in patients under valproic acid treatment and in the elderly. This may be due to the lower carnitine and/or acetylcarnitine found in these patients. Patients with controlled seizures had normal carnitine and acetylcarnitine levels. Further studies are necessary in order to conclude if the uncontrolled bipolar disorder could be the cause of higher carnitine and/or acetylcarnitine levels.

## 1. Introduction

L-Carnitine is a ubiquitous molecule derived from the amino acids lysine and methionine; its homeostasis is maintained through dietary intake and endogenous formation. Skeletal and myocardial muscles are the main consumers of L-carnitine and contain the highest depots in the body. Uptake in these tissues is dependent upon active and saturable transport: Organic Cation Transporter 2 (OCTN2), this transport being accountable for intestinal and renal reabsorption also [1, 2]. L-Carnitine pool comprises free carnitine (CAR) and esterified derivatives or acylcarnitines (ACYLCAR), formed by means of carnitine acyltransferases in several tissues. Under normal metabolic conditions acetylcarnitine (ALCAR) is the major representative of the acyl group and participates in anabolic and catabolic pathways of cellular metabolism [3]. CAR and short-chain ACYLCAR are excreted by the kidneys and reabsorbed in as much as 99% [2, 4]. Although 99% of CAR is found intracellularly, the relationship between serum ACYLCAR and CAR is highly sensitive to intramitochondrial metabolic alterations [5]. It has been proposed by several authors [6] that to determine if there is a real CAR deficiency ACYLCAR/CAR ratio is a good biomarker. In adults under normal conditions, ACYLCAR/CAR ratio should be between 0.1 and 0.4; values above 0.4 indicate CAR deficiency [7].

CAR and ALCAR perform major functions in the body. Both of them are involved as cofactors for transport of long chain fatty acids through the mitochondria membrane. The maintenance of the mitochondrial acyl-CoA/CoA ratio has been signaled as a crucial role of CAR, owing to the fact that many enzymes involved in the citric cycle, gluconeogenesis, the urea cycle, and fatty acid oxidation are regulated by the aforementioned ratio [5]. Trans esterification of acyl-CoA esters to CAR by the action of carnitine acetyltransferase (CAT) restores intramitochondrial free CoA and releases mainly ALCAR which can then be used for acetyl-CoA synthesis. Hence, the actions of CAT, CAR, ALCAR, and CoA pools are in close relationship [2, 3].

ALCAR and CAR are able to cross the blood brain barrier and are present in the brain in high concentration, reaching nervous areas where the linked acetyl group may be delivered. The possibility of providing acetyl groups makes ALCAR able to maintain the intramitochondrial salvage pathways, to reactivate coenzyme A, to reduce peroxidation and intracellular malonyl aldehyde levels, to act as a scavenger, and to contribute to neurotransmitter synthesis due to the structural affinity to acetyl-choline [4].

Ammonia is the main toxic electrolyte produced by our body as a byproduct of protein catabolism in the intestine and muscle. Its elimination is a fine equilibrium which may be altered by many factors, such as liver and renal pathologies, age, and medication [8–11]. Ammonia is mainly eliminated through the urea cycle which has a prerequisite: the formation of carbamoyl phosphate by means of carbamoyl phosphate synthetase I (CPS I). This step controls urea formation rate, placing CPS I regulation as a key factor in ammonia elimination. This enzyme is allosterically regulated by N-acetylglutamate (NAG) which is synthesized using acetyl-CoA and glutamate [12] and it can be inhibited by some drugs [13, 14]. Any factor altering this cycle could result in hyperammonemia. With the exception of valproic acid (VPA), other anticonvulsant drugs do not lead to elevated ammonia levels [15]. In a study carried out by our research group with patients under valproic acid (VPA) treatment, we found higher ammonia levels related to higher VPA and 4-en-VPA concentrations, the latter being a toxic metabolite able to inhibit CPS I [16]. Moreover, reports in the literature associated the use of VPA with decreased carnitine levels [17].

Carnitine is important for energy production in skeletal muscles and there seems to be a negative correlation between advancing age and muscle carnitine levels. The information regarding CAR status in the elderly is rather controversial as it was stated by most authors that decrease in biosynthesis together with impaired reabsorption favors lower levels of CAR with life decline [18].

Some central nervous system (CNS) pathologies such as uncontrolled epilepsy and Alzheimer can be the cause of high levels of ammonia in blood [19, 20].

The aim of this study was to analyze if the use of VPA, the age of the patients, or certain CNS pathologies can cause CAR and/or ALCAR deficiency leading to increased ammonia levels.

## 2. Materials and Methods

### 2.1. Subjects

Subjects were divided into three groups: Group A: epileptic patients under phenytoin (PHT) monotherapy; Group B: patients with bipolar disorder under VPA treatment; Group C: elderly patients (65 years or older).

 Group A: thirty-one adult patients (15 men, 16 women), mean age 46.2 years (range 18–55), were included. CAR and ACYLCAR profiles and ammonia concentrations in blood were determined.
 Group B: twenty-eight patients treated with VPA (15 men, 13 women), mean age 45.7 years (range 18–55) were included. Plasma VPA, blood CAR and ACYLCAR profiles, and ammonia blood concentrations were determined.
 Group C: forty-one patients (21 men, 20 women), mean age 73.8 years (range 65–85), were recruited. CAR and ACYLCAR profiles and ammonia blood concentrations were determined.

Comedication and comorbid conditions were evaluated in order to include the patients in the different groups. Patients with renal impairment and liver dysfunction were excluded.

All subjects came to the Therapeutic Drug Monitoring Service in the morning after an overnight fasting period. For CAR and ACYLCAR determination a few drops of blood from a finger prick were collected onto filter paper cards and dried.

For VPA (Group B) and ammonia (Groups A, B, and C) determination, a blood sample was withdrawn prior to morning dose by arm venipuncture, collected and divided into two tubes containing EDTA and heparin for ammonia and VPA determinations, respectively. Due to stability reasons, EDTA tubes were immediately taken by refrigerated transport to the laboratory for immediate determination of ammonia (within 30 minutes from blood sampling). For VPA determination blood samples were centrifuged and plasma was stored at −20°C until analysis.

The study protocol was approved by the Institutional Ethics Review Committee of the Faculty of Chemistry,* Universidad de la República*. All subjects participating in the study received a leaflet with the protocol details and signed a consent form before entering the study.

### 2.2. VPA Analysis

Plasma VPA determination was performed by a previously published validated high performance liquid chromatography method (HPLC) with minor modifications [21].

Thirty microliters of internal standard (octanoic acid, OCT) was added to 1.0 mL of plasma. A Phenomenex® Luna CN 5 *μ*m (150 mm × 4.6 mm) column was used as stationary phase. The mobile phase was a mixture of potassium phosphate monobasic 40 mM pH 3.4/acetonitrile (90/10) pumped with a flow rate of 1.5 mL/min. The column compartment was kept at 36°C, and the wavelength detection was 210 nm. Under these conditions the retention times of analytes were 4.9 and 6.8 min for VPA and OCT, respectively. The HPLC method was linear between 1.1 mg/L and 133 mg/L. Within-day and between-day precisions for low, intermediate, and high concentrations were below 15%. Accuracies at the same concentration levels were within 92 and 108%.

### 2.3. CAR and ACYLCAR Analysis

CAR and ACYLCAR were quantified using liquid chromatography-tandem mass spectrometry (LC-MS/MS). Extraction was performed on 3.2 mm filter paper disks punched out from dried blood spot specimens using 100% methanol solution containing the internal standards. The internal standards used were the Cambridge isotopes internal standards NSK sets A and B which contained the following stable isotopes (acylcarnitines: d9-C0, d3-C2, d3-C3, d3-C4, d9-C5, d3-C8, d9-C14, and d3-C16). The extracted samples were derivatized with 3 N butanolic HCl at 65°C and finally reconstituted with acetonitrile/water (50 : 50) solution containing 0.02% formic acid (mobile phase). Samples were analyzed with HPLC-MS/MS (Dionex-ABSiex 3200 triple quadrupole) using precursor-ion of 85 *m*/*z*.

### 2.4. Ammonia Determination

Blood ammonia concentration was determined by Cobas (enzymatic automated assay, c311, Roche Laboratories). Hyperammonemia is a condition characterized by elevation in the serum level of ammonia above 94 *μ*g/dL.

### 2.5. Data Analysis

Mean ammonia, CAR, and ALCAR concentrations were compared among the three patients' groups to establish if VPA, pathology, or age may modify their levels. ACYLCAR/CAR ratios were computed and compared also.

For the patients treated with VPA, the anticonvulsant concentration was correlated with CAR, ALCAR, and ammonia levels. Ammonia, CAR, and ALCAR levels were compared, to determine if ammonia increase may be caused by deficiency of CAR and/or ALCAR.

Ammonia, CAR, and ALCAR were correlated with age to study if life decline influenced their levels.

All statistical analyses were carried out using SPSS (version 17.0 for Windows). Means were compared using Student's *t*-test for independent variables. All *p* values were two-sided, using *α* = 0.05 as the reference standard for determining the significance.

## 3. Results

As shown in Table 1, mean ammonia concentration (SD) in Group A was 61.7 (27.3) *μ*g/dL falling within the reference ammonia levels of our laboratory (25–94 *μ*g/dL). CAR, ALCAR concentrations, and ACYLCAR/CAR ratio were 37.8 (8.6) *μ*mol/L, 11.5 (3.0) *μ*mol/L, and 0.35 (0.15), respectively. These values do not differ from the reference values of our laboratory or the reported ones in the literature [2, 18, 22]. None of the patients in this group presented hyperammonemia.

**Figure 1:**
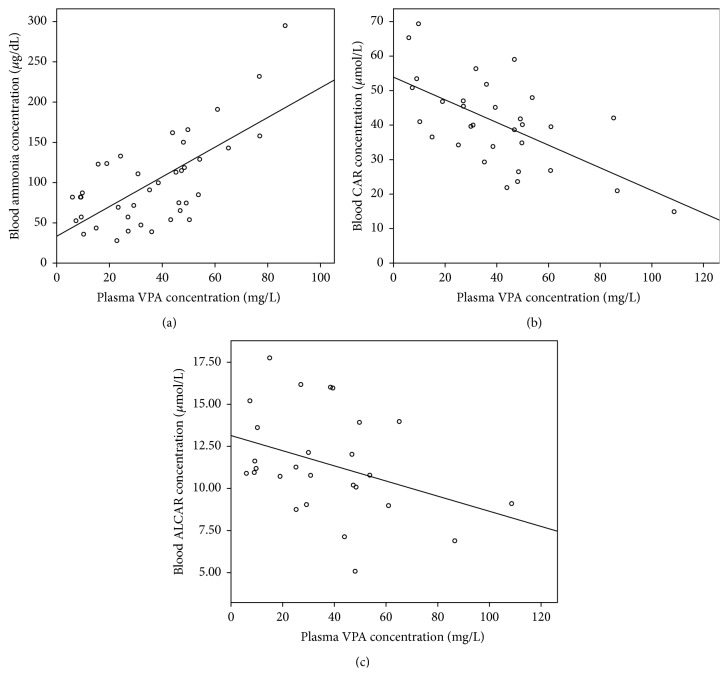
Relationship between plasma VPA concentration and (a) blood ammonia concentration (*y* = 1.84*x* + 33.41, *R* ^2^ = 0.467, and *p* < 0.025); (b) blood CAR concentration (*y* = −0.328*x* + 53.90, *R* ^2^ = 0.380, and *p* < 0.0001) and (c) blood ALCAR concentration (*y* = −0.045*x* + 13.14, *R* ^2^ = 0.133, and *p* = 0.062).

In Group B, 8 patients presented hyperammonemia, representing 28.6% of the subjects treated with VPA. All the patients with hyperammonemia remained asymptomatic. In this group, ammonia level (105.2 ± 57.2 *μ*g/dL) was significantly higher (*p* < 0.005) than in Group A. CAR and ALCAR levels did not show any differences between the two groups; nonetheless, when patients were divided according to their ammonia level and compared within Group B, patients with hyperammonemia presented CAR (34.9 ± 11.4 *μ*mol/L) and ALCAR (9.9 ± 3.0 *μ*mol/L) concentrations significantly lower (*p* < 0.05 and *p* < 0.002, resp.) than patients with normal levels of ammonia. Patients with hyperammonemia presented ALCAR levels significantly lower than patients of Group A and there was a trend towards lower CAR levels also. The previous finding was corroborated by a positive correlation between VPA concentration and ammonia level (*p* < 0.025), whereas VPA correlated negatively with CAR (*p* < 0.0001) and there was a trend towards lower ALCAR levels, as shown in Figures 1(a), 1(b), and 1(c) respectively. Patients with hyperammonemia in Group B presented an ACYLCAR/CAR ratio of 0.44 (0.13) significantly different (*p* < 0.001) from Group A, 0.35 (0.15), showing that ALCAR and/or CAR deficiency may be present in patients with hyperammonemia treated with VPA. These results are in accordance with other studies that propose CAR depletion in patients with long term VPA treatment [23]. There was a significant difference (*p* < 0.05) in VPA plasma concentrations in Group B between patients with hyperammonemia and patients with normal ammonia levels, 44.9 (29.8) versus 30.1 (20.3) mg/L, respectively.

CAR levels in subjects of Group C (50.1 ± 18.9 *μ*mol/L) were significantly higher (*p* < 0.001) than in younger adults of Group A (37.8 ± 8.6 *μ*mol/L). On the other hand, mean ALCAR concentration showed a significant difference (*p* < 0.001), in favor of younger adults; see Table 2. Elderly patients showed higher ammonia levels of 82.1 (35.6) *μ*g/dL than epileptic patients of Group A, *p* < 0.05. The iatrogenic cause of this ammonia increase was excluded after thorough study of their pharmacotherapy; thereby etiology should be found elsewhere. Regarding the comparison of ACYLCAR/CAR between Groups A and C, a significant difference was found showing elderly patients lower ratios (*p* < 0.025).

**Figure 2:**
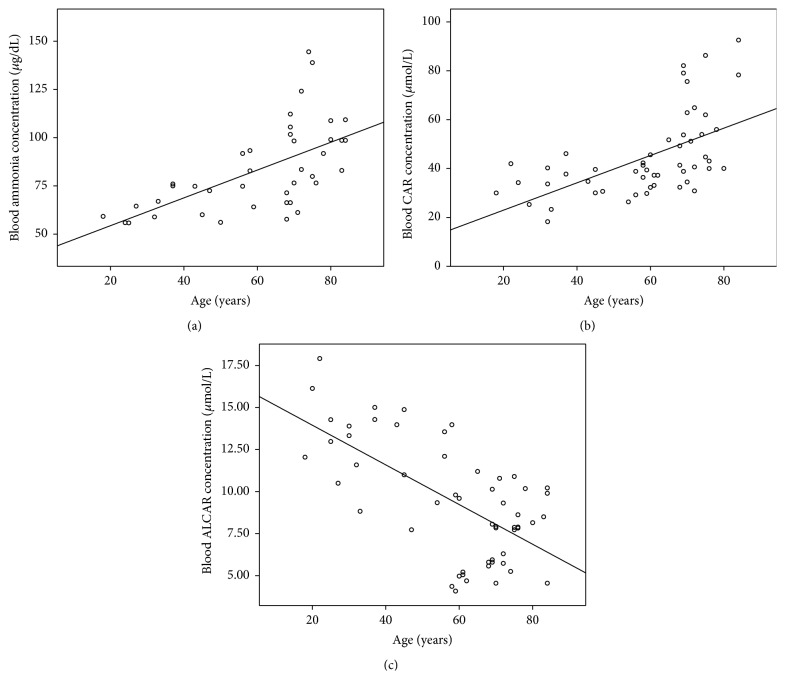
Relationship between age and (a) blood ammonia concentration (*y* = 0.721*x* + 39.95, *R* ^2^ = 0.367, and *p* < 0.001); (b) blood CAR concentration (*y* = 0.585*x* + 32.65, *R* ^2^ = 0.327, and *p* < 0.001) and (c) blood ALCAR concentration (*y* = −0.118*x* + 16.31, *R* ^2^ = 0.411, and *p* < 0.001).

Ammonia, CAR, and ALCAR correlations with age are shown in Figures 2(a), 2(b), and 2(c), respectively (Groups A and C). As can be observed a positive correlation was found between ammonia versus age (*p* < 0.01) and CAR versus age (*p* < 0.001) whereas a negative one was found between ALCAR and age (*p* < 0.001).

**Figure 3:**
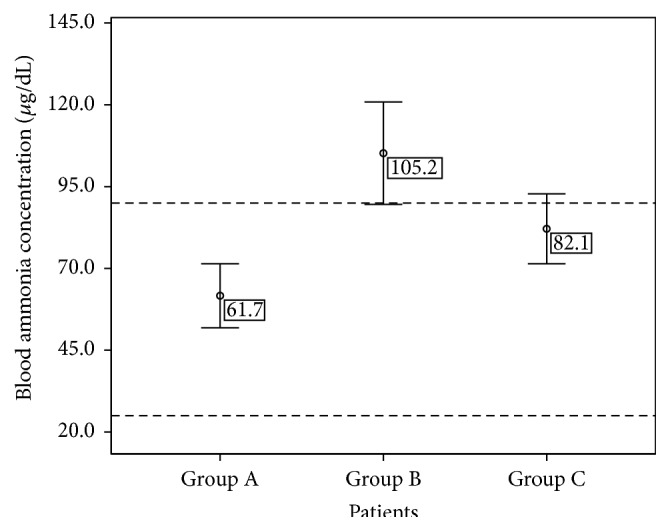
Mean ammonia levels (±SD) in epileptic patients under phenytoin monotherapy (Group A), in patients with bipolar disorder treated with valproic acid (Group B), and in elderly patients (Group C). Dashed lines indicate the normal ammonia range in blood (25–94 *µ*g/dL).

Figure 3 shows mean ammonia levels in the three groups. Patients of Groups B and C show ammonia levels significantly higher than patients in Group A.

In the three groups, some patients had more than one VPA, CAR, and ALCAR determinations whereas in some patients ALCAR could not be determined.

## 4. Discussion

The results obtained in Group A showed that controlled seizures, as referred to in the Introduction, did not influence CAR, ALCAR, and ammonia levels or CAR availability as defined by the ACYLCAR/CAR ratio lower than 0.4.

VPA-associated hyperammonemia is related to CAR deficiency [24–27]. This could lead to less *β*-oxidation and as a result lower acetyl-CoA production; if that were the case not only CAR levels but also ALCAR ones would be affected, owing to the fact that acetyl-CoA may be considered as a precursor of the latter [28]. In our patients of Group B with hyperammonemia, lower CAR and ALCAR levels were found in comparison to Group A. Noteworthy, low CAR concentrations may involve ammonia increase due to the fact that acetyl-CoA is used for NAG synthesis as explained in the Introduction. It should be considered that if VPA metabolism through *β*-oxidation is impaired as a result of CAR deficiency, the *ω*-oxidation route is favored leading this pathway to the production of 4-en-VPA, which explained our previously reported findings [16].

Surprisingly, when comparing CAR and ALCAR levels in patients of Group B without hyperammonemia to Group A, significant higher concentrations were found in the former subgroup (*p* < 0.025 and *p* < 0.001, resp.). Could the disease itself (bipolar disorder) be the cause of this difference? CAR and ALCAR especially, as it is better absorbed and more able to cross the blood brain barrier [29], are emerging agents with growing evidence supporting their use in several neuropsychiatric disorders mainly depression [30, 31]. Although ALCAR's exact mechanism of action in treatment of depression is still not clear, animal and cellular models suggest that its neuroplasticity effect, membrane modulation, and serotonergic and possibly dopaminergic activities regulation could play an important role as possible action mechanisms [32]. However, its benefits must be thoroughly studied. A case has been reported [33] in which ALCAR administration precipitated psychosis in a patient with bipolar disorder. Perhaps higher levels of CAR and/or ALCAR are present in patients with bipolar disorders not stabilized with VPA yet. VPA might have as additional benefit in the treatment of bipolar disorder, the decrease of abnormal CAR and/or ALCAR levels. No studies in the literature were carried out measuring CAR and ALCAR levels in patients with bipolar disorders. So, further studies are necessary in order to conclude if the uncontrolled pathology itself could be the cause of the higher CAR and ALCAR levels.

Aging is associated with higher oxidative stress and ALCAR lower levels have been pointed out as one of the causes [11, 34]. ALCAR biosynthesis is dependent upon CAR entrance into the cell. OCTN2, the main transporter mediating CAR entrance into the cell is present in heart, liver, kidneys, intestine, and skeletal muscle [35]. Elderly subjects may present decreased levels of OCTN2 [18, 36]. This condition could influence CAR entrance to the cell and as a result its metabolism to ALCAR. Moreover, ALCAR renal reabsorption could also be impaired. This could lead to higher circulating CAR levels and lower ALCAR formation which is in agreement with the results obtained in this study.

In that scenario, ALCAR deficiency is placed at the spotlight, strengthened by some studies reporting hyperammonemia reversion with ALCAR administration in patients with minimal hepatic encephalopathy [37, 38]. ALCAR has also shown encouraging results in the treatment of degenerative brain diseases where cognitive functions are involved [39, 40] and in reversing the hyperammonemia in patients with Alzheimer [20].

In spite of the usefulness of ACYLCAR/CAR ratio in other populations, in the elderly this ratio can be misleading. As serum CAR levels are augmented and ALCAR (the main component of ACYLCAR) is diminished there can be a false impression that CAR deficiency is not present, when in fact circulating CAR levels are high but tissue concentrations remain low because of impaired transport.

Both Groups B and C showed significantly higher ammonia levels in comparison with Group A. Nevertheless, as explained before, a different etiology underlies the two situations. In patients treated with VPA, CAR levels are diminished and ALCAR follows along, whereas in elderly patients CAR circulating levels are increased but ALCAR biosynthesis is diminished and responsible for the higher values of ammonia observed in this population. There is evidence, as stated previously, that ALCAR is better than CAR at improving oxidative stress markers [41] and together with the correction of ammonia shown in patients with Alzheimer disease, the amelioration of hyperammonemia symptoms, both in patients treated with VPA and in the elderly, may have the same solution: ALCAR administration.

## 5. Conclusions

Patients under PHT treatment with controlled seizures showed no decrease in CAR and ALCAR levels and thus no increase in ammonia concentrations was observed.

In patients treated with VPA, CAR depletion followed by ALCAR decrease could be responsible for the increase in the ammonia levels. On the other hand, in the elderly population, serum CAR could be increased due to impaired access to tissues which in turn could result in ALCAR decrease. This last fact could lead to ammonia impaired elimination. Perhaps higher ammonia levels and ALCAR deficit could be responsible for the cognitive and neurodegenerative diseases found in the elderly. ACYLCAR/CAR ratio is not a good biomarker for all populations.

Although ALCAR arises as a promising agent to control ammonia levels, it should be used with caution in patients with bipolar disorders, the use of CAR being preferable.

## Figures and Tables

**Table 1:** Mean ± SD ammonia, CAR, and ALCAR levels and ACYLCAR/CAR ratio in patients with PHT (Group A) and in patients with VPA (Group B). Mean plasma VPA concentrations of patients in Group B are also shown.

	Ammonia ± SD (*μ*g/dL)	VPA ± SD (mg/L)	CAR ± SD (*μ*mol/L)	ALCAR ± SD (*μ*mol/L)	ACYLCAR/CAR ± SD
Group A	61.7 ± 27.3		37.8 ± 8.6	11.5 ± 3.0	0.35 ± 0.15
Group B	105.2 ± 57.2		39.8 ± 13.0	12.6 ± 5.5	0.45 ± 0.13
*p* value	0.005		NS	NS	0.0005
Normal values in healthy individuals	25–94		19–59	>9.8	0.1–0.40
Group B					
With hyperammonemia	140.0 ± 47.7^a^ *p* < 0.001	44.9 ± 29.8	34.9 ± 11.4	9.9 ± 3.0^b^ *p* < 0.025	0.44 ± 0.13^c^ *p* < 0.005
Without hyperammonemia	54.6 ± 19.7	30.1 ± 20.3	44.5 ± 10.3^b^ *p* < 0.025	17.4 ± 5.7^a^ *p* < 0.001	0.47 ± 0.11

^a,b,c^Significantly different from the corresponding value in Group A.

**Table 2:** Mean ± SD ammonia, CAR, and ALCAR levels and ACYLCAR/CAR ratio in epileptic patients under phenytoin treatment (Group A) and in elderly patients (Group C).

	Ammonia ± SD (*μ*g/dL)	CAR ± SD (*μ*mol/L)	ALCAR ± SD (*μ*mol/L)	ACYLCAR/CAR ± SD
Group A	61.7 ± 27.3	37.8 ± 8.6	11.5 ± 3.0	0.35 ± 0.15
Group C	82.1 ± 35.6	50.1 ± 18.9	7.9 ± 2.7	0.29 ± 0.12
*p* value	<0.05	<0.0001	<0.025	<0.025
